# Total elbow replacement in England: a protocol for analysis of National Joint Registry and Hospital Episode Statistics data

**DOI:** 10.1186/s13018-024-04903-9

**Published:** 2024-08-30

**Authors:** Zaid Hamoodi, Adrian Sayers, Michael R. Whitehouse, Amar Rangan, Lianne Kearsley-Fleet, Jamie C. Sergeant, Adam C. Watts

**Affiliations:** 1 Centre for Epidemiology Versus Arthritis, Centre for Musculoskeletal Research, Manchester Academic Health Science Centre, University of Manchester, Oxford Road, Manchester, UK; 2 Upper Limb Unit, Wrightington Hospital, Wrightington, Wigan and Leigh Teaching Hospitals NHS Foundation Trust, Wigan, UK; 3 Musculoskeletal Research Unit, Translational Health Sciences, Bristol Medical School, Bristol, UK; 4 National Institute for Health Research Bristol Biomedical Research Centre, University Hospitals Bristol NHS Foundation Trust and University of Bristol, Bristol, UK; 5 South Tees Hospitals NHS Foundation Trust, Middlesbrough, UK; 6 Health Sciences and Hull York Medical School, University of York, York, UK; 7 Centre for Biostatistics, School of Health Sciences, Faculty of Biology, Medicine and Health, Manchester Academic Health Science Centre, University of Manchester, Manchester, UK; 8 Health Research Institute, Edge Hill University, Ormskirk, UK

**Keywords:** Elbow, Replacement, National Joint Registry, Protocol, Incidence

## Abstract

**Introduction:**

Primary total elbow replacement (TER) services in England are being restructured with the goal of centralising care to specialised centres. It is important to monitor the impact of this service redesign. This protocol outlines an intended analysis to provide detailed descriptions of the patients who are receiving primary TER, where and by whom TER is being performed, and what the current surgical practices for TER are in England before the reconfiguration.

**Methods:**

This analysis will use the National Joint Registry (NJR) elbow dataset and link it with NHS England Hospital Episode Statistics-Admitted Patient Care (HES-APC). It will include eligible patients from the start of the NJR elbow dataset in April 2012 to December 2022. The main objective is to determine the incidence of TER in England. Age-sex standardised rates will be calculated for groups including different ethnicities, and socioeconomic backgrounds, using the mid-year population data provided by the Office for National Statistics. This planned analysis will summarise patient characteristics such as age, sex, body mass index (BMI), hand dominance, American Society of Anaesthesiologists (ASA) grade, indication for TER, socioeconomic status, and patient co-morbidities. It will also examine implant fixation type, classification, brand/type, and changes over time in implant types used in England. Additionally, it will explore the characteristics and volume of the surgeons and hospitals providing primary TER services, including the grade of the primary surgeons, funding source for surgery, and admission type. The analysis will cover the number of procedures performed by surgeons and hospitals annually in England and in each region of England. Finally, the planned analysis will summarise the elective wait time, postoperative length of stay, and any serious adverse events or re-admissions within 30 and 90 days after the TER.

**Discussion:**

This protocol describes the first deep dive analysis into the NJR elbow dataset to describe the incidence of TER surgery in England and the characteristics of patients who are receiving it. This analysis will summarise current primary TER practices in England before service reconfigurations. The impact of reconfiguration can be monitored by comparing future practice to the outcomes from this study.

*Trial registration* ClinicalTrials.gov, NCT06355011. Registered 02 April 2024, https://clinicaltrials.gov/ct2/show/NCT06355011.

**Supplementary Information:**

The online version contains supplementary material available at 10.1186/s13018-024-04903-9.

## Introduction

Total elbow replacement (TER) is an established treatment of painful elbow conditions including inflammatory arthritis, osteoarthritis, trauma sequalae, and in the treatment of complex distal humerus fractures [1]. Despite its established role, the number of TERs performed each year is much lower than other joint replacements such as hip, knee, and shoulder replacements [2]. In England and Wales, the number of TERs performed yearly between 2012 and 2022 ranged between 258 and 463 [2]. In 2018, the British Elbow and Shoulder Society (BESS) reported the average number of TERs performed per surgical unit to be two to three and that 73 surgeons performed one TER in 2016 [3]. This was a cause for concern, as there are studies in the lower limb and shoulder replacement surgery reporting higher volumes, by surgeons and hospitals, are associated with lower revision rates [4–8]. In addition, it has been reported that specialised centres and surgeons who perform a higher number of TERs have a lower risk of revision, although the quality of this evidence was judged to be very low in a recent systematic review [9–11].

In 2015, the Getting It Right First Time (GIRFT) national programme was introduced to improve medical care within the NHS by reducing unwarranted variations in outcomes [12]. GIRFT sought to rationalise the delivery of orthopaedic care within the NHS in England, addressing cost and efficiency. One aspect GIRFT targets is to centralise the provision of low volume procedures, such as TER, to specialised centres [12]. BESS and GIRFT have collaborated to produce guidelines and recommendations focused on the delivery of primary and revision elbow replacement [3]. Discussions have been held to reduce the number of centres providing primary and revision TER. The perceived benefit is to increase in the average number of TERs performed per surgeon and unit with concentration of resources, experience, and expertise, and to increase training opportunities to improve patient outcomes [12].

Whilst there are theoretical benefits to service changes, with some evidence from other countries that rationalisation of services can improve outcomes, it is important to monitor the effect of service redesign [13]. The purpose of this protocol is to outline the intended analysis of the TER procedures carried out in England, with a specific focus on (1) which patients are receiving TER surgery, (2) where and by whom TER surgery is undertaken, and (3) current surgical practices for TER, in England.

## Method and analysis

The findings and methodology of this study will be reported in accordance with the REporting of studies Conducted using Observational Routinely-collected health Data (RECORD) statement [14].

### Source of data

In this analysis data from the National Joint Registry (NJR) will be used. The primary purpose of NJR is to collect data on joint replacements to provide timely warnings of issues relating to patient safety [15]. In doing so, the NJR collects high-quality data that is commonly used in orthopaedic research [15]. The NJR elbow dataset will be the primary data source and it will be linked with data from the NHS England Hospital Episode Statistics-Admitted Patient Care (HES-APC) dataset to incorporate data that are not collected by the NJR.

The NJR started collecting elbow replacement procedures in April 2012. It is currently compulsory for all NHS and independent hospitals in England, Wales, Northern Ireland, the Isle of Man, and the States of Guernsey to submit elbow replacement surgery data, including primary and revision surgery, to the NJR [16]. The NJR collects data using a collection tool called the Minimum Data Set (MDS) form, which is usually filled out by a clinician at the time of surgery. Information regarding the implants used is usually completed by administration staff by including the implant codes (usually stickers that are attached to each implant) in the MDS form. All the information from the MDS forms is entered directly into the NJR data entry system locally by the hospital where the procedure was performed. When the NJR started collecting elbow replacement procedures in April 2012, version 5 of the MDS (MDSv5) was in use. The MDS has been updated twice to version 6 in November 2014 and version 7 in June 2018. Data collection using MDS version 8 started in June 2023, and the changes made in MDSv8 will not impact this project. The changes are summarised in Supplementary File 2.

There is currently a live automated data quality audit process to address any potential missing procedures, which started in June 2020 [17]. A collaborative audit between the NJR, British Orthopaedic Trainee Association (BOTA), British Orthopaedic Association (BOA), BESS and The Royal College of Surgeons of England (RCSEng) showed the completeness of the TER dataset to be 93% and the accuracy to be 98%. This audit included all TER procedures from the start of data collection in April 2012 until the live automated audit started. High completeness of the target population limits selection bias and increases the generalisability of results [18].

The HES-APC dataset contains details about inpatient admissions funded by the NHS, including patients in independent hospitals funded by NHS trusts. All patients undergoing TER surgery are admitted to hospital, and their data will be included in the HES-APC. Each NHS trust collects data while the patient receives treatment. The data are usually collated locally by clinical coders using the patient medical records and then submitted to NHS England. Once collated, NHS England processes and assesses the quality of the data and the yearly HES datasets are released for secondary use, including research. HES includes demographic, clinical, and administrative information which can be used to assess the practices and trends of joint replacement surgery. These include information such as co-morbidities, socioeconomic status, length of hospital stay, and ethnicity [19].

The data linkage of HES-APC to the NJR will be performed as part of an existing agreement between the NJR and NHS England. Access to the data is facilitated under NJR permissions. The data linkage will be performed by applying seven linkage methods using a combination of matched NHS numbers, Local patient ID, date of birth, year of birth, gender, and/or matched care provider (Supplementary File 1). Procedures that are matched based any of those linkage methods will be included, but for a procedure to match, the HES-APC hospital episode start date must be the same or before the NJR operation date whilst the NJR operation date must be before or the same as the HES-APC episode end date.

### Patient and public involvement

The Patient and Public Involvement and Engagement (PPIE) members at Wrightington hospital were consulted and involved in the study objectives and methodology.

### Ethics

The NJR Research Committee approved this study [20]. The NJR supports public health surveillance and wider clinical decision-making and holds pseudonymised data that are anonymous to the researchers who use it. The NHS Health Research Authority tool guidance dictates that the secondary use of such data for research does not require approval by a research ethics committee [21]. Patients consented to inclusion in the NJR according to standard practice, with permission under the Health Service (Control of Patient Information) Regulations, otherwise referred to as Section 251 support [22].

### Participants

**Fig. 1:**
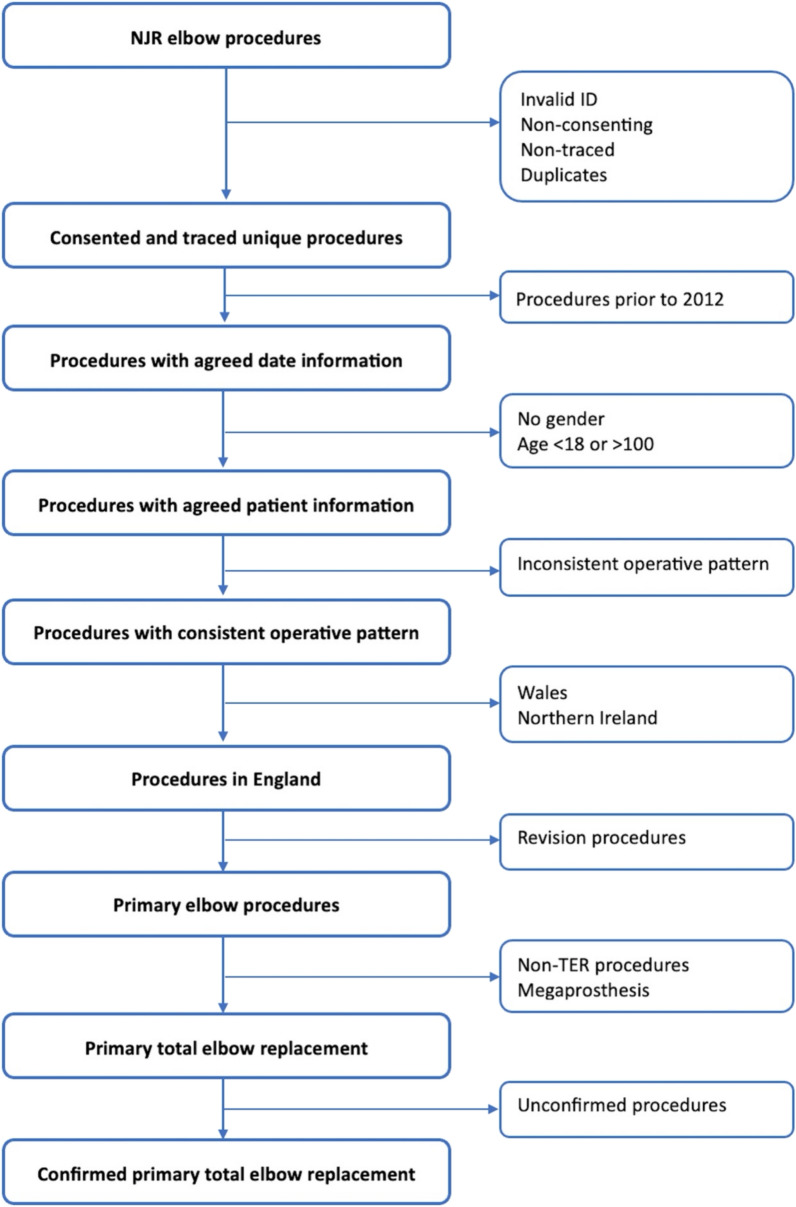
Flow diagram for the unlinked National Joint Registry (NJR) elbow replacement dataset

**Fig. 2:**
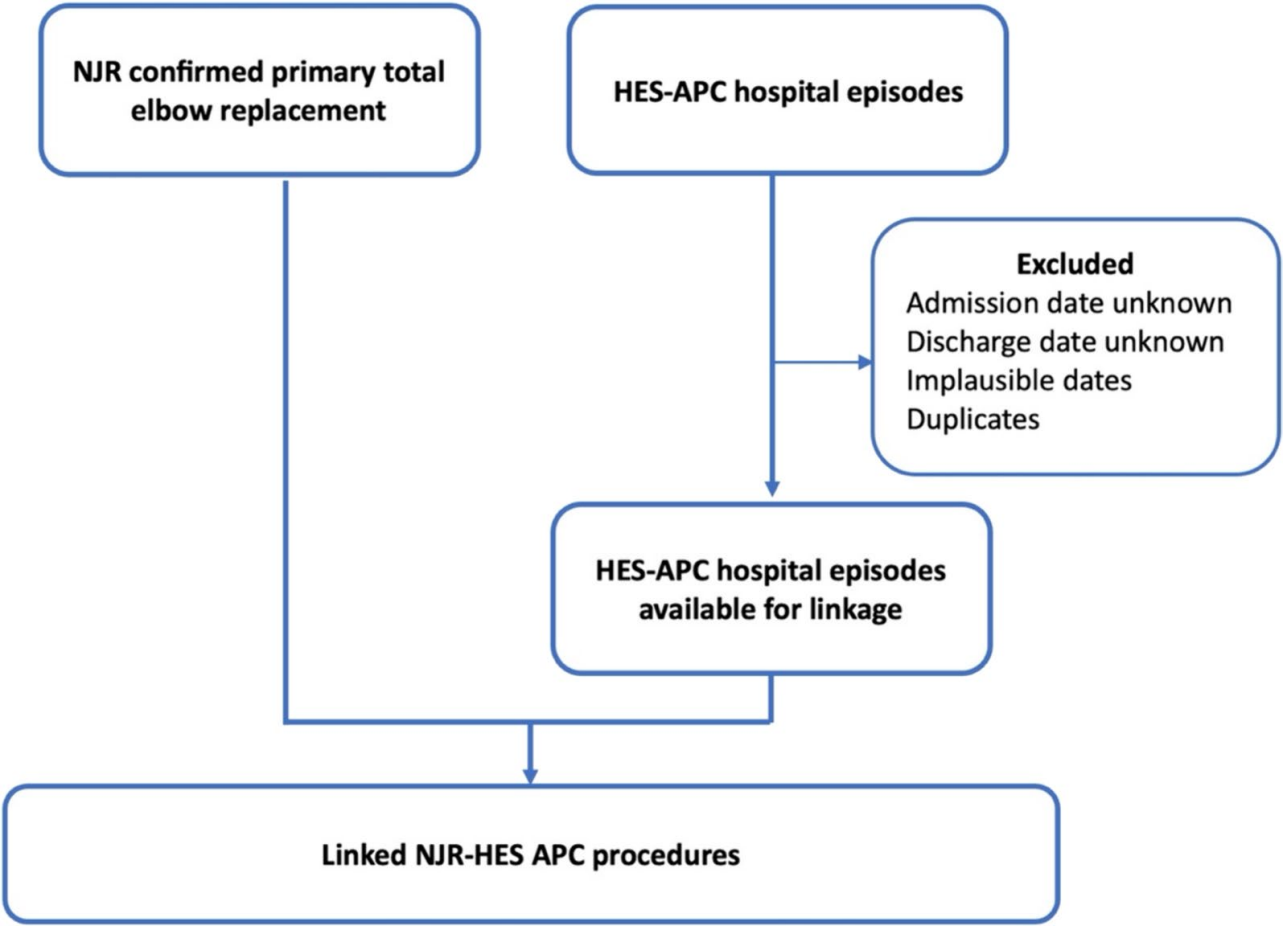
Flow diagram for the linked National Joint Registry (NJR) and NHS England Hospital Episode Statistics-Admitted Patient Care (HES-APC)

All patients aged 18–100 years old with a primary TER on the NJR elbow dataset from the start of data collection on the 1st of April 2012 to the 31st of December 2022 will be included. Patients are excluded if they did not consent for their data to be used for research purposes, if it is impossible to trace them after surgery, if their ID numbers are invalid, or if the surgery was not performed in England. Several steps will be undertaken to confirm that the included cases are primary TER procedures. This will include (1) ensuring all operative patterns are consistent (2) confirming all reported procedures on the NJR matches the implant components submitted. Unconfirmed procedures and procedures with inconsistent operative patterns (i.e. a sequence of operations where the primary operation is not the first operation in the sequence or where there are multiple primary operations recorded for the same joint) will be excluded from the analyses. The data preparation process and exclusion of non-eligible procedures is summarised in Fig. 1. Eligible NJR procedures will then be linked to the available HES-APC data which includes all patients’ hospital episodes from 23rd of October 1996 to 31st of March 2022. Hospital episodes with invalid ID numbers, implausible dates, unknown discharges dates, and duplicate episodes will be excluded (Fig. 2).

### Variables

Most of the data from the NJR and HES-APC can be extracted directly but some of the variables used in this study will be derived using the available data and the methods of how this will be performed are highlighted in this section.

#### Patient related variables

Patient related variables will be extracted from the NJR and HES-APC data. The list of the patient related variables and their data source is displayed in Table 1. Age will be derived from date of birth; socioeconomic status will be derived from postcode as an Index of Multiple Deprivation quintile (2015 version) [23]; and co-morbidities will be derived from the ICD-10 (international classification of diseases, 10th revision) codes reported by HES-APC. The list of co-morbidities for each patient will include all co-morbidities available in the HES-APC hospital episodes up to and including the primary TER. The co-morbidity list will be used to calculate the original Charlson Comorbidity Index [24] and a revised version of Charlson Index using an updated weights which are calibrated using English data due to differences in coding practice and hospital patient population characteristics [25]. If BMI is not reported, then the value will be derived from the height and weight variables if they are reported. The indication for surgery in will be reported as five categories: acute trauma, inflammatory, trauma sequalae, osteoarthritis and other. Procedures that are reported as Essex Lopresti on the NJR will be included in the acute trauma category if the injury is acute or in the trauma sequelae if the injury is chronic. The decision whether the Essex Lopresti injury is chronic or acute will be decided by the admission type and duration of surgery wait on the HES-APC data. Non-elective admissions and elective admissions with less than two weeks from decision for surgery to the date of surgery will be considered as acute injuries. Procedures reported as avascular necrosis will be added to the “other” category. Multiple indications for surgery can be selected on the MDS forms. In this study the likely primary indication will be selected based on a hierarchy agreed by the research team. Acute trauma will be the primary diagnosis if it is selected followed by trauma sequelae, inflammatory arthritis, osteoarthritis, and then “other” category. Ethnicity was categorised into six categories based on the Office of National Statistics (ONS) Ethnic group classification 6a [26]. If ethnicity is missing from the hospital episode admission for TER surgery, ethnicity will be established from other hospital episodes admissions for that patient where available.

**Table 1:** Patient related variables to be included in the planned study

Variable	Dataset	How will data be presented	Method of measurement
Age	NJR	Continuous and age categories	Derived from the date of birth and date of surgery
Sex	NJR	Categorical: Male/Female/Indeterminate	Documented directly using a specified list on MDS collection form
BMI	NJR	Continuous and categorical: Underweight/Normal/Overweight/Obese/Morbidly obese	Documented directly or derived from the weight and height of the patient
Dominant hand	NJR	Categorical: Yes/No/Unknown	Documented directly using a specified list on MDS collection form
ASA	NJR	Categorical: ASA1-ASA5	Documented directly using a specified list on MDS collection form
Indication for surgery	NJR	Categorical: (1) Trauma/Elective (2) Acute trauma/inflammatory/Trauma Sequalae/Osteoarthritis/Other	Documented directly using a specified list on MDS collection form
Socioeconomic status	NJR	Categorical: Indices of multiple deprivation quintiles (2015 version)	Derived from the postcodes and reported as index of deprivation quintiles
Ethnicity	HES	Categorical: Asian, Asian British, or Asian Welsh/Black, Black British, Black Welsh, Caribbean or African/White/Mixed or Multiple ethnic groups/Other ethnic group/Does not apply (students and schoolchildren living away during term-time)	Documented in patient medical records. Classification is based on the ONS group classification 6a [26]
Co-morbidities	HES	(1) Categorical: Acute MI/CHF/PVD/Cerebrovascular Disease/Dementia/COPD/Rheumatoid Disease/Peptic Ulcer/Mild liver disease/Diabetes/Diabetes + Complications/Hemiplegia or Paraplegia/Renal disease/Cancer/Moderate/Severe liver disease/Metastatic Cancer/AIDS/Depression/Anxiety/Osteoporosis (2) Original Charlson Comorbidity Index Hospital (3) Charlson Comorbidity Index Hospital with revised weights	Documented in patient medical records and extracted by admin team at the time of discharge and reported using ICD-10 codes The Charlson Comorbidity Index will be derived from pre-existing condition recorded on HES-APC data using ICD-10 codes

*ASA* American Society of Anaesthesiologists, *BMI*: Body Mass Index, *MI* myocardial infarction, *CHF* congestive heart failure, *PVD* peripheral vascular disease, *COPD* chronic obstructive pulmonary disease

#### Implant related variables

Implant related variables will be derived from the NJR data only. They are based on classifying each implant in the NJR data. In this study, the classification used for the NJR annual report will be used [27]. There are different combinations of the Latitude implant reported by the NJR 20th annual report. In this study Latitude will be classified as Latitude Legacy, Latitude EV, or Latitude mix (i.e., EV and Legacy components). The list of the implant related variables and how they will be measured is shown in Table 2.

**Table 2:** Implant related variables to be included in the planned study

Variable	Dataset	How will data be presented	Method of measurement
Fixation type	NJR	Categorical: Cemented/Uncemented	Derived from implant codes on the MDS form
Implant classification	NJR	Categorical: Linked/Unlinked	Derived from implant codes on the MDS form and the list of components (e.g. if a linkage component was submitted with a likable implant)
Implant type	NJR	Categorical: Coonrad-Morrey/Discovery/Latitude (Legacy, EV, Mix)/GSB III/MUTARS/Nexel/IBP	Derived from implant codes on the MDS form
If RHR was used	NJR	Categorical: Yes/No	Derived from implant codes on the MDS form and the list of components

*NJR* National Joint Registry, *RHR* radial head replacement

#### Surgeon and hospital related factors

Most of the surgeon and hospital variables used in this study are not directly reported but will be derived from the available data (Table 3). The NJR includes pseudonymised codes to represent the surgeon who performed the surgery and the hospital where the surgery was performed. This data can be used to calculate the surgeon’s and hospital’s volume. In this project, the annual number of TERs will include all TERs from the 1st of January to the 31st of January of that year. The year 2012 will be excluded from this analysis because data is only available from April 2012.

**Table 3:** Surgeon and hospital related variables to be included in the planned study

Variable	Dataset	How will data be presented	Method of measurement
Funding	NJR	Categorical: NHS/Independent sector	Documented directly using a specified list on MDS collection form
Grade of primary surgeon	NJR	Categorical: Consultant/Other	Documented directly using a specified list on MDS collection form
Surgeon volume	NJR	Number of TERs performed by a surgeon per year by surgeons	Derived from pseudonymised codes representing the surgeon in charge of patient care. It represents the number of TERs performed from 1st of January to the 31st of December of each year
Hospital volume	NJR	Number of TERs performed by a hospital per year by surgeons	Derived from pseudonymised codes representing the hospital where TER was performed It represents the number of TERs performed from 1st of January to the 31st of December of each year
Regional volume	NJR	Number of TERs per year by surgeons by region	Derived from the hospital where TER was performed. It represents the number of TERs performed from 1st of January to the 31st of December of each year
Duration of elective wait	HES	Number of days waiting	Derived from the date which it was decided to admit the patient and actual admission date
Post-operative duration of stay	HES	Number of inpatient days following surgery	Derived from the date of surgery and date of discharge
Elective admission type	HES	Categorical: General admission/Day case admission	Documented in patient medical records

The duration of elective wait and the length of post-operative stay will be derived from the HES-APC data. To establish the duration of elective wait, the difference in days between the date on which it was decided to admit the patient and the date of surgery will be calculated. The length of post-operative stay will be derived from the difference in days between the date of the TER and discharge date. Elective admissions will be classified into general admissions or day case admissions. Day case admission will be derived from the admission method and spell duration variables on HES-APC. For an elective procedure to be classified as day case it must be an elective admission and has a spell duration of 0 days.

### Outcome

The primary outcome in this proposed study will be the number and/or the rate of provision of primary TER. Secondary outcomes will include the duration of elective wait and post-operative duration of stay measured in days. Current trends will be described by reporting the outcomes on annual basis.

Serious adverse events (SAE) within 30 days and 90 days from the index TER will also be reported. SAE will be defined as any severe medical complications leading to hospital admission, including pulmonary embolism, myocardial infarction, lower respiratory tract infection, acute kidney injury, urinary tract infection, cerebrovascular events, and all-cause death. SAE will be extracted from the HES-APC data and identified using ICD-10 codes.

### Statistical analyses

Descriptive analysis will be performed for all included variables. Frequencies and proportion will be used to summarize categorical variables. The distribution of continuous variables will be assessed using histograms. It is likely that some of the continuous data, such as surgeons’ and hospitals’ volume, will be skewed, therefore, continuous variables will be reported using the median and interquartile range (IQR). The analyses will include summary of all the included population, stratified analysis for elective and acute trauma population, and analysis for each year from 2012 to 2022. The number of procedures performed by surgeons and hospitals will be summarised for the whole population and for each region in England and the results will also be reported on annual basis.

TER rates for different sexes, age categories, socioeconomic status categories and different ethnic groups will be reported. The rates of primary TER per 100,000 persons will be calculated by dividing the number of procedures in the NJR elbow dataset by the corresponding mid-year population estimates published by the Office for National Statistic (ONS). Sensitivity analysis will be performed using the census estimate from 2021. The population estimates by ethnic group reported by ONS will be used to estimate the rates of TER between different ethnicity groups. Age and sex standardised TER rates for each IMD group will be reported. Statistical analyses will be performed using Stata version 18 (StataCorp LP, USA).

## Discussion

This protocol describes the first deep dive analysis into the National Joint Registry (NJR) elbow dataset to describe the incidence of Total Elbow Replacement (TER) surgery in England and the characteristics of patients who are receiving it. By linking the National Joint Registry (NJR) with the Hospital Episode Statistics-Admitted Patient Care (HES-APC) data of NHS England, additional analysis can be conducted that was previously not possible in this group of patients. This includes examining patient ethnicity, comorbidities, post-operative length of stay, and readmissions after surgery. This study will summarise current primary TER practices in England before service reconfigurations. The impact of reconfiguration can be monitored by comparing future practice to the outcomes from this study. The study may be limited due to the method used to collect HES data, which involves data extraction from non-standardised and largely unstructured paper records.

A limitation of using the elbow dataset of the joint registry is the need for clinical outcomes (e.g., the range of movement) or patient-reported outcomes. Although those outcomes are important in elbow surgery, none were available from either of the datasets we plan to use in this analysis. It would be desirable to have those outcomes collected pre- and post-TER surgery, and their collection by joint registries should be considered in the future.

### Supplementary Information

Below is the link to the electronic supplementary material.

Supplementary Material 1.

Supplementary Material 2.

## Data Availability

No datasets were generated or analysed during the current study.

## Abbreviations

BESS: British Elbow and Shoulder Society
BOA: British Orthopaedic Association
BOTA: British Orthopaedic Trainee Association
GIRFT: Getting It Right First Time
HES-APC: Hospital Episode Statistics-Admitted Patient Care.
IQR: Interquartile ranges
MDS: Minimal dataset
NJR: National Joint Registry
ONS: Office for National Statistic
RCSEng: Royal College of Surgeons of England
SAE: Serious adverse events
TER: Total elbow replacement
