# Supplementary material for: Total elbow replacement in England: a protocol for analysis of National Joint Registry and Hospital Episode Statistics data
Source: J Orthop Surg Res. 2024 Aug 30;19:526. doi: 10.1186/s13018-024-04903-9 (PMC11363632; doi:10.1186/s13018-024-04903-9)
Supplement: Supplementary file 1 — Supplementary Material 1. [file 13018_2024_4903_MOESM1_ESM.docx]

Supplementary File 1

Data linkage method

| Linkage method | NHS numbers | Local patient ID | NJR trust code HES-APC Code of Provider (procode3) | Date of Birth | Year of birth | Gender | HES-APC episode start date is the same or before the NJR operation date whilst the NJR operation date is before the HES-APC episode end date |
| --- | --- | --- | --- | --- | --- | --- | --- |
| 1 | **Matched** |  |  |  |  |  | **Matched** |
| 2 | **Matched** |  | **Matched** |  |  |  | **Matched** |
| 3 |  | **Matched** | **Matched** |  |  |  | **Matched** |
| 4 |  |  | **Matched** | **Matched** |  |  | **Matched** |
| 5 |  |  | **Matched** | **Matched** |  | **Matched** | **Matched** |
| 6 | **Matched** |  |  |  | **Matched** |  | **Matched** |
| 7 | **Matched** |  |  |  | **Matched** | **Matched** | **Matched** |

Table 1 Methods of linkage between the National Joint Registry (NJR) and NHS England Hospital Episode Statistics-Admitted Patient Care (HES-APC)
