# Supplementary material for: Total elbow replacement in England: a protocol for analysis of National Joint Registry and Hospital Episode Statistics data
Source: J Orthop Surg Res. 2024 Aug 30;19:526. doi: 10.1186/s13018-024-04903-9 (PMC11363632; doi:10.1186/s13018-024-04903-9)
Supplement: Supplementary file 2 — Supplementary Material 2. [file 13018_2024_4903_MOESM2_ESM.docx]

**Supplementary File 2**

**The changes in NJR data collection between different MDS version**

The MDS version 5 used was used between April 2012, and November 2014. MDS Version 6 was used between November 2014 and June 2018 and MDS was used between June 2018 June 2023. It is important to note that not all hospitals changed to the newer MDS version immediately.

| **Variable** | **MDS** | **Changes in MDS** | **How will changes be addressed in this study** |
| --- | --- | --- | --- |
| Sex | 5-7 | None |  |
| BMI | 7 | Only started data collection in MDSv7 | 1. Use available BMI data 2. Calculate BMI from height and weight if available |
| Dominant hand | 5-7 | None |  |
| ASA | 5-7 | None |  |
| Funding | 5-7 | None |  |
| Grade of surgeon | 5-7 | None |  |
| Assistant | 5-7 | None |  |
| Side | 5-7 | None |  |
| Indication | 5-7 | MDSv5 had a failed humeral hemiarthroplasty as an indication for primary TER. This has changed to revision TER since MDSv6. | Primary TER performed humeral hemiarthroplasty will be excluded as this is a revision procedure |
| Surgical Approach | 5-7 | None |  |
| Anaesthetic type | 5-7 | MDSv5 and MDSv6 had four categories: General/ Regional – Nerve block / Regional Epidural / Regional – Spinal  MDSv7 has only two categories general/ regional nerve block | Not reported in this study |
| Minimally invasive | 5-7 | None |  |
| Computer guided surgery | 5-7 | None |  |
| Chemical VTE prophylaxis | 5-7 | Addition of Factor Xa Inhibitor (e.g. Rivaroxaban/Apixaban) to MDSv6 and MDSv7 | Not reported in this study |
| Mechanical VTE prophylaxis | 5-7 | None |  |
| Humeral bone grafting | 5-7 | Type of Bone graft was added in MDS 7 | Not reported in this study |
| Ulna bone grafting | 5-7 | Type of Bone graft was added in MDS 7 | Not reported in this study |
| Intra-operative complications | 5-7 | None |  |
| Fixation type | 5-7 | None |  |
| Approach | 5-7 | None |  |
